# Experiencing authenticity to environmentally responsible behavior: Assessing the effects of perceived value, tourist emotion, and recollection on industrial heritage tourism

**DOI:** 10.3389/fpsyg.2022.1081464

**Published:** 2022-12-15

**Authors:** Tao Zhang, Chen Wei, Lingyue Nie

**Affiliations:** Faculty of International Tourism and Management, City University of Macau, Macau, Macao SAR, China

**Keywords:** industrial heritage, authentic experience, perceived value, emotion, tourists’ recollection, environmentally responsible behavior

## Abstract

As an important part of cultural heritage, industrial heritage has important cultural, historical and economic values. There are many studies on authentic experience in industrial heritage sites, but the studies on responsible behavior of authentic experience to environment of industrial heritages are still lacking. Therefore, this study takes Nanjing Quarry Park as the research object and S-O-R theory as the theoretical basis, after combing the literature on five aspects of authentic experience, perceived value, emotion, tourists’ recollection and environmentally responsible behavior, the authors made questionnaires and conducted field research, the authentic data of tourists’ authentic experience in Nanjing Quarry Park were obtained by questionnaire and analyzed, and the research model of responsible environmental behavior of authentic experience in industrial heritage scenic spots was constructed. In conclusion, this study found that authentic experience and perceived value can induce positive and negative emotions in tourists, and then produce memories, and act on environmentally responsible behavior. Emotion plays a part of mediating role in authentic experience, perceived value, recollection and environmentally responsible behavior. Positive emotion and perceived value have significant positive effects on recollection and tourists’ responsible behavior, while negative emotion and perceived value have significant negative effects on recollection, there was no significant negative effect on responsible behavior of environment. Authentic experience is an important antecedent of emotion.

## Introduction

Industrial heritage tourism is a new endeavor in the tourism that encompasses both heritage tourism and industrial civilisation. The growth of industrial heritage tourism can not only satiate tourists who are “searching for novelty and fulfilling curiosity,” but it can also satiate tourists who are “seeking knowledge and reflecting on the past” (Li, 2009; Wang, 2014; Lu and Qiu, 2018; Liang, 2020; Liu, 2020; Shao, 2020). As industrial heritage tourism grows, the local ecology will undoubtedly face some threats from the flood of visitors. As a result, researchers are starting to pay attention to tourists’ environmentally responsible behavior, which is seen as essential to the growth of industrial heritage tourism sites in a sustainable manner (Yuan, 2008; Xi, 2013; Dou, 2016; Yang, 2019; Zhang, 2021). Enjoyment is the primary motivation for travel, and emotion are what make travel memorable (Xie, 2006). The essence of industrial heritage, is found in “authenticity” (Ruan, 2008).

**Figure 1:**
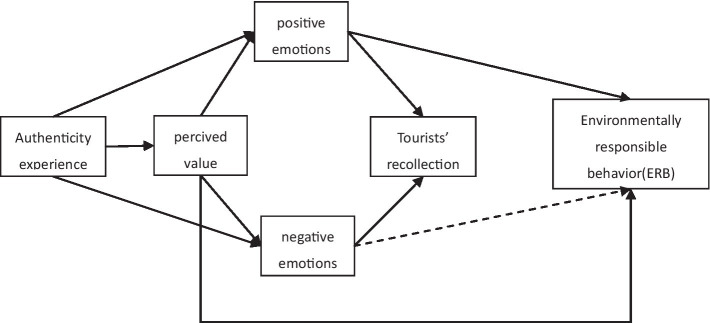
Conceptual model.

Authenticity experience has become a critical concept in industrial heritage tourism research. In fact, the core of industrial heritage is authenticity experience (Ruan, 2008). Environment, souvenirs, themes/stories, mood, relationships, values, and individual aspects all have an effect on how authentic an experience is (Xie and Qu, 2014). Authentic experience has a positive effect on emotional experience (Du et al., 2018). Obviously, authenticity has been considered as a significant driver of customers who validate emotional experience in tourism activities (Wang, 2020). Cui (2019) identified authenticity experience has substantial practical implications for boosting the worth and allure of industrial heritage tourism areas and fostering the sustainable growth of tourism sites. Liu and Ma (2010) demonstrated that clients’ negative emotional has a increase effect on their inclination to misbehave. Walker and Chapman (2003) reported that emotional helps improve visitors to protect the environmental behavior. Xia and Chen (2015) demonstrated that the perceived value of ecological tourists can affect their environmentally responsible behavior. Prior studies identified that in order to figure out the environmental problems of industrial heritage, the breakthrough point should start with the authenticity, perceived value, emotion and recollection. There is no complete framework to explain how visitors can generate environmentally responsible behavior through authenticity experience. Therefore, this study employed the S-O-R theory and explored the relationship between authenticity experience, perceived value, emotion, tourists’ recollection and environmentally responsible behavior. Building the mechanism model of the influence of authenticity experience on environmental responsible behavior. Emotion plays a part of mediating role in authentic experience, perceived value, recollection and environmentally responsible behavior. Positive emotion and perceived value have significant positive effects on recollection and tourists’ responsible behavior, while negative emotion and perceived value have significant negative effects on recollection. Improve the relevant research on industrial heritage tourism, In order to further enhance the attractiveness of industrial heritage tourism, Provide targeted improvement suggestions for management, To give tourists a better travel experience in the future, To enhance the environmental responsible behavior of tourists, It provides relevant reference and basis for the operation, management and sustainable development of Nanjing Tangshan Quarry Park (Figure 1).

## Literature review

### S-O-R theory

S-O-R theory includs three basic elements: stimulus, organism, and response. Wang (2020) defined Stimulus (S) received by an individual will help individual develop internal states (Organism), elicit consequent responses (R). He and Gong (2010) used the S-O-R theory to build a model of how online shopping experiences affect customers’ behavior and came to the conclusion that sensory experience and emotional experience has a positive effect on customers’ emotions, emotion and trust has a positive effect on customers’ behavior. Employing the S-O-R model, Wei and Wan (2020) found perceived quality and perceived risk are intermediary variables in an online virtual community and the influence of online retail corporate social responsibility on consumers’ purchase willingness from the perspective of consumers’ online shopping. Zhang (2021) proposes a conceptual model between virtual brand community experience based on the S-O-R model, community relationship quality, and purchase intention, and finds that there is a positive impact on the information experience, interactive experience, entertainment experience and purchase intention of virtual brand community through empirical research. Community satisfaction, community trust, and community commitment to the quality of community relationships also have a positive impact on purchase intent. The SOR theory in the tourist are flourish in new stage. Huang (2012) examined the S-O-R theory in the context of cultural parks to examine the effect of environmental factors on organisms (O), the conclusion that particular circumstances and employee performance can affect behavior. It appears that the S-O-R theory has gained better applicability in various sectors, including tourism.

### Authentic experience

Authenticity, also known as the “veracity,” originally used to describe the veracity of religious history, was later applied to the preservation of the industry heritage tourism (Ruan, 2008). The Venice Charter introduced the “authenticity” in 1964, and since then, authenticity has the touchstone of the world industrial heritage (Gazzola, 1964). Nara Document on Authenticity, published in 1994, outlined a comprehensive framework for authenticity and placed emphasis on the value of fostering and maintaining industrial heritage (Stovel, 2008). Researchers have discovered that it more difficult for tourists to feel the history and cultural simply by tourism motivation and authenticity (Gu et al., 2018). There are not many domestic studies on tourism motivation, authenticity and satisfaction. The research’s focus on the heritage sites’ architecture, cultural activities, souvenirs, and food.

### Perceived value

Perceived value is one of the key ideas that research in the service business. Related studies have started to concentrate on perceived value in the tourism industry since the middle of the 1990s. Jackson (1985) introduce the idea of perceived value, emphasized that perceived value is the proportion of effort and recognition. Zeithaml (1988) reported that perceived value is the visitor’s overall appraisal of the products or service. Woodruff (1997) revealed that the core of the perceived value is discuss the perceived advantages and perceived disadvantages. Zeithaml (1988) divided it into two dimensions-material cost and psychological cost. Carothers and Adams (1991) based his division of the visitor perceived value dimension into two dimensions – tangible product and intangible product. Sheth and Newman (1991) suggested that four dimensions of perceived value: social value, emotional value, functional value, and cognition value. Solakis et al. (2022) demonstrated that value co-creation in the constext of hospitality services, including dialogue, access, risk, and transparency, could increase and enhance tourists’ perceived value. This suggests that tourists’ authentic experience of industrial heritages may lead to positive feelings and therefore enhance their perceived value of industrial heritages.

### Emotions

Tourist emotion is a key concept in the fields of visitor behavior and marketing (Liu and Jin, 2015). Previous researchers demonstrated that in tourism, emotions divided into two main sections: negative and positive (Le and Dong, 2013). Positive emotions are a reflection of enthusiastic, upbeat, and positive people feel, whereas negative emotions are a reflection of disgust, guilt, fear, and tension. Hosany (2012) study that tourists are influenced by both positive emotional reactions such as happiness, surprise and fondness, as well as equally causal variables such as anger, disappointment and contempt in negative emotions. Xie (2006) proposed a bipolar affective model of tourism experience: pleasure-pain. Meng (1985) argued that positive emotions and negative emotions can have different effects on human behavior. Mitas et al. (2012) discovered that tourists feel that having satisfying while traveling will foster positive emotions. Lin et al. (2014) discovered that tourists’ emotions will have a positive effect on tourists’ attitudes toward and evaluation of tourism. In conclusion, positive and negative emotions are significant that have the power to both positively and negatively influence tourists’ behavior.

### Tourists’ recollection

A key factor in tourism is the tourists’ recollection. Johar et al. (2006) reported that recollectionis a process of study knowledge. Schacter et al. (1993) defined that recollection is an ability that person to bring past experiences into the current consciousness. Bower (1970) regarded as there are two types of recollection: short-term recollection and long-term recollection. Long-term recollection is the storage of information based on the “retelling” of the primary recollection, whereas short-term recollection is the main recollection formed by the action of a stimulus. Bettman (1979) revealed that recollection can be separated into perceived recollection and recollective recollection. The aforementioned information leads to the conclusion that recollection is crucial. Clawson and Knetsch (2013) suggested that only when the traveler can recollect or remember the past of the journey is the experience significant and meaningful. Thus, tourists’ recollection is vitally important in tourism.

### Environmentally responsible behavior

Borden and Schettino (1971) first proposed ‘environmentally responsible behavior. Sivek (1987) defined that environmentally responsible behavior as actions taken by individuals or groups to protect the environment. Sivek and Hungerford (1990) expanded this definition, they reported that environmentally responsible behavior should be a series of actions taken by individuals or groups to reduce the use of natural resources in order to promote the sustainable use of natural resources. Cheng et al. (2013) defines environmentally responsible behavior as actions taken by individuals or groups to preserve and protect the environment. Li and Wang (2011) defined that environmentally responsible behavior is the behavior of visitors that satisfies the requirements of environmental friendliness and resource conservation. Fan and Qiu (2014) research that the relationship between the place identity and environmentally responsible behavior. Dou (2016) defined tourist environmentally responsible behavior as the behavior made by tourists that has the least negative effect on the local ecological environment and actively promotes the sustainable use of natural resources in the tourism place.

Environmental attitudes, emotions, experiences, ethics, satisfaction, perceived value, and place attachment are among the psychological factors that affect environmentally responsible behavior (Hines et al., 1987; Bamberg and Möser, 2007; Steg and Vlek, 2009; Klöckner, 2013; Fan and Qiu, 2014). Contextual factors that influence environmentally responsible behavior mainly include: role influence, information campaigns, policies and regulations, material inducements, social norms, and behavioral costs (Stern, 2000; Darby, 2006; Linden et al., 2006; Steg and Vlek, 2009; Sun et al., 2012; Wang and Wang, 2015).

## Theory and hypotheses

### The relationship between emotions, perceived value and authenticity

Zheng et al. (2020) reported that tourism can elicit positive or negative emotions. For instance, visiting World Heritage Sites (WHS) can elicit positive emotions like pride, happiness and confidence. In consumer theory, negative emotion of consumers might result a series of negative impacts. Such as customer complaints, replacement service providers (e.g., Enrique Bigné et al., 2008). While negative emotions are seen undesirable and can result in a variety of negative outcomes, positive emotion are thought to have positive consequences (Nawijn and Biran, 2019). Therefore, we propose the following hypothesis:

*H1*: authentic experience has a positive effect on visitors’ perceived value.

*H2*: authentic experiences has a positive effect on tourists’ positive emotions.

*H3*: authentic experience has a negative effect on tourists’ negative emotions.

*H4*: perceived value has a positive effect on increases visitors’ positive emotions.

*H5*: perceived value has a negative effect on tourists’ negative emotions.

### The relationship between tourists’ recollection, emotions and environmentally responsible behavior

Recollection and how people interpret information are both influenced by emotion, which is a critical factor (Kuhl, 1986). Emotions to some extent influence visitors’ recollection (Babin et al., 1998; Lee et al., 2008; Su and Swanson, 2017). Emotions include positive and negative emotion, and when they are stimulated emotionally, they can help create salient memories (Dewhurst and Parry, 2000; Kensinger and Corkin, 2003; Kim, 2014).Visitors’ emotions of happiness or dissatisfaction generate mental imprints that can influence visitor recollection (Rubin and Kozin, 1984; Kozak, 2001; Talarico and Rubin, 2003; Arora and Singer, 2006; Thomsen and Hansen, 2009; Zeithaml et al., 2012). Positive emotions experienced when traveling might leave tourists with long-lasting effects. Similarly, negative emotions can cause negative memories (Su et al., 2020). According to several research, people base their decisions on their sentiments and emotions (Mehrabian and Russell, 1974; Donovan and Rossiter, 1982). Different behaviors are significantly influenced by emotions (Westbrook, 1987; Nyer, 1997). Positive emotions, according to Romani et al. (2013), mediate CSR and advocacy actions, which affect people’s behavior. Izard (1977) and Jang and Namkung (2009) contend that emotions mediate perceptions of conduct and quality. Visitors visiting natural areas can experience the comfort of the natural environment and get knowledge about ecological vulnerability, according to Ballantyne and Packer’s (2011) research. Through education, visitors are able to alter their views and actions. Su et al. (2020) contend that tourists’ emotions have an effect on how they behave in terms of protecting the environment. In contrast, negative emotions cause visitors’ environmental responsibility to drop. Positive emotions boost tourists’ environmental responsibility. As a result, it therefore proposes eight hypotheses:

*H6*: positive emotions has a positive effect on tourists’ recollection.

*H7*: negative emotions has a negative effect on tourists’ recollection.

*H8*: positive emotions has a positive effect on influence ERB.

*H9*: negative emotions has a negative effect on ERB.

*H10*: The effects of perceived value and ERB is mediated by positive emotions.

*H11*: The effects of perceived value and ERB is mediated by negative emotions.

*H12*: The effects of perceived value and visitor recollection are mediated by positive emotions.

*H13*: The effects perceived value and visitor recollection are mediated by negative emotions.

### Perceived value and environmentally responsible behavior

A number of empirical studies research the relationship between environmentally responsible behavior and perceived value. The reciprocity criterion suggests that if the benefits outweigh the cons for tourists, they are more likely to have a positive travel experience and are therefore more likely to be motivated to protect the environment. Russell and Russell (2010) argued that perceived value is essentially the concept of weighing the pros and cons. Chiu et al. (2014) reported that tourists’ perceived value influences in environmentally responsible behavior. Huang et al. (2017) and other studies also came to the same conclusion. Consequently, the following hypotheses are proposed:

*H14*: Perceived value has a positive effect on ERB.

## Methodology

### The research site and measurement

The case site of this study is the “Nanjing Tangshan Quarry Park,” which is 40 km east of Nanjing city in Jiangsu province, China. It is situated in the renowned Tangshan Hot Spring Resort and has a total size of around 40 acres. This study’s questionnaire is divided into two main sections. The respondents’ basic demographic information, such as gender, age, education level, and monthly personal income, is covered in the first section. The second part is the question items for each of the main variables, derived from previous relevant studies and appropriately adapted to specific tourism scenarios. The authentic experience for this study was modified from the studies of Zatori et al. (2018) and Zhang et al. (2008) and designed as five question items. Visitor perceived value was modified in conjunction with studies by McIntosh and Prentice (1999), among others. Positive emotions and negative emotions were modified primarily from the studies of Zheng et al. (2020) and Nawijn and Biran (2019) and designed as three items each. Visitor recollection was modified primarily from studies such as Kyle et al. (2005) and Kim (2010). Environmentally responsible behavior was mainly referencing the study by Huang et al. (2017). This portion consists of 24 questions, each with a five-point Likert scale, with 1 = strongly disagree and 5 = strongly agree.

### Data collection and analysis

Six days of data collection were spent on February 1–5, 2022, and February 1–15, 2022, with 312 questionnaires issued, of which 303 were valid and had a 97.1% questionnaire efficiency. The majority of the research was done at the dining area, children’s play area, and exit of Nanjing Tangshan Mining Park. The surveys were given out randomly. The questionnaire distributors-a total of five teachers and graduate tourist management students-were uniformly instructed to comprehend the objectives of the study and the requirements of the research before executing the questionnaire distribution in order to ensure the quality of data collecting. The analytical tools of SPSS 24.0 and AMOS 25.0 were employed in data analysis. According to a descriptive analysis of the valid questionnaires’ demographic data, the respondents were made up of the following: the proportion of males (56.1%) was slightly higher than that of females (43.9%), and the majority of tourists were between the ages of 25 and 34 (29.4%) and 35 and 44 (28.1%), which was consistent with the observations made during the study. Young friends were most frequently seen traveling in groups of two or three, and the educational level was mainly concentrated in the 25–34 (29.4%) and 35–44 (28.1%) age brackets. The biggest percentage of visitors visited the park more than twice (45.9%), showing that the majority of tourists were inclined to return to the park. The highest percentage of visitors’ *per capita* monthly income was between 5,000 and 10,000 RMB (31.4%).

## Results

### Measurement model-reliability and validity analyses

First, the data were examined for reliability using SPSS 24.0 to determine the internal consistency in each dimension (Table 1), and it was discovered that the authentic experience, perceived value, positive emotion, negative emotion, visitor recollection, and environmentally responsible behavior had respective Cronbach coefficients of 0.870, 0.876, 0.712, 0.666, 0.708, and 0.857, and the overall Cronbach coefficient was 0.937, which satisfied the internal consistency requirements and the value of Corrected Item-Total Correlation (CITC) at above 0.5 level. Therefore, the level of reliability was achieved for the dimensions of the investigated constructs in the research.

**Table 1:** Results of confirmatory factor analysis (*n* = 303).

Constructs/Items	Estimate	SE	C.R.	AVE	CR
*Authenticity experience*					
The quarry park is well preserved	0.725			0.5534	0.861
Build the scenic spot with virtually no distortion	0.767	0.086	12.485		
Added facilities are consistent in layout and arrangement with the intact environment very well	0.746	0.08	12.159		
The quarry park arouses feelings of authentic industrial history and culture	0.739	0.081	12.048		
Destination’s resources are original	0.742	0.082	12.105		
*Perceived value*					
Scenic area to provide a comprehensive service facilities	0.736			0.5712	0.8693
Provide the most effective service to the tourist	0.723	0.078	12.202		
There are lots of interesting souvenirs to buy	0.757	0.075	12.808		
Activities to suitable for different groups of people	0.78	0.076	13.205		
With a distinct environment theme	0.781	0.083	13.225		
*Positive emotion*					
When I was visiting this destination, I felt enjoyment	0.808			0.612	0.8254
When I was visiting this destination, I felt happy	0.783	0.064	14.477		
When I was visiting this destination, I felt relaxed					
*Negative emotion*	0.755	0.07	13.853		
When I was visiting this destination, I felt anger	0.955			0.642	0.8405
When I was visiting this destination, I felt boring	0.706	0.062	12.306		
When I was visiting this destination, I felt disgust	0.718	0.062	12.502		
*Tourists’ recollection*					
I had wonderful memories about this experience	0.748			0.5672	0.7976
I will not forget my experience on this tourism	0.78	0.079	13.424		
The memory of that day still haunts me	0.732	0.079	12.551		
*Environmentally responsible behavior*					
I learned a lot of environmental protection knowledge	0.714			0.5232	0.8458
I will discussion with friends about environment	0.708	0.086	11.439		
I do not ruin destination’s environments	0.741	0.091	11.956		
I do not step into non-open areas	0.741	0.088	11.953		
I would like to attend environmental protection activities	0.712	0.08	11.512		

Next, confirmatory factor analysis (CFA) with AMOS 25.0 revealed that for convergent validity, the factor loadings of all test items were >0.5, the combined reliability (CR) ranged from 0.79 to 0.86, and all were >0.6; the average variance extracted (AVE) all met the criterion of 0.5, indicating that the model had good basic fitness and high structural validity. The average variance extracted (AVE) was 0.5, demonstrating the model’s strong structural validity and good basic fitness. The goodness-of-fit indices were reported at *χ*^2^/df = 1.302, *p* < 0.001, RM-SEA = 0.032, CFI = 0.980, TLI = 0.978, IFI = 0.981, reaching a satisfactory level of model fit for the measurement model in accordance with the criteria stipulated. Calculating composite reliabilities proved adequate internal consistency of the items. The reliability of each construct was above the suggested value of 0.70 (Hair et al., 2010). Items factor loadings <0.5 were deemed as insignificant and prone to cross-loading (Byrne, 2010).

The square root of the average variance extracted from each variable (diagonal bold numbers) is generally regarded as being greater than the correlation coefficient between any two variables in the following analysis of the discriminant validity (see Table 2). This indicates that the discriminant validity between the latent variables is good. In this study’s variables, the square root of the arithmetic is greater than the correlation coefficient, as shown in Table 2, which suggests good discriminant validity.

**Table 2:** Correlations and discriminant validity.

Constructs	AE	PV	PE	NE	TR	ERB
Authentic experience (AE)	0.744					
Perceived value (PV)	0.488**	0.756				
Positive emotions (PE)	0.574**	0.615**	0.782			
Negative emotions (NE)	0.126*	−0.134*	−0.061	0.801		
Tourists’ recollection (TR)	0.598**	0.685**	0.728**	−0.126*	0.753	
Environmentally responsible behavior (ERB)	0.531**	0.690**	0.668**	−0.045	0.620**	0.723

* Correlation is significant at the 0.05 level (2-tailed).

** Correlation is significant at the 0.01 level (2-tailed).

### Structure equation model

The parameters of the structural equation model were estimated using the maximum likelihood method in AMOS 25.0 and all fit indicators (*χ*^2^/df = 1.302, RM-SEA = 0.032, CFI = 0.980, TLI = 0.978, IFI = 0.981) met the criteria. The hypothesis model was tested and the results showed (as in Table 3) that: the direct effect of tourists’ negative emotions on environmentally responsible behavior did not reach a significant level (*p* = 0.595 > 0.05) and thus hypothesis H9 was rejected; the authentic experience had a significant positive effect on perceived value, positive and negative emotions tourists (*p* > 0.05) and H1, H2, and H3 were supported; perceived value had a significant positive effect on positive emotions, environmentally responsible behavior (*p* > 0.05) all had a significant positive effect, supported by H4 and H12; positive emotions had a significant positive effect on both tourist recall and environmentally responsible behavior (*p* > 0.05), supported by H6 and H8; perceived value had a significant negative effect on negative emotions, and negative emotions on tourist recall (*p* > 0.05, coefficient negative), supported by H5 and H7.

**Table 3:** Structure parameter estimates.

Hypothesis path	Estimate	SE	C.R.	Value of *p*	Result
PV ← AE	0.662	0.081	8.176	***	Support
PE ← AE	0.413	0.062	6.636	***	Support
NE ← AE	0.336	0.091	3.694	***	Support
PE ← PV	0.47	0.056	8.365	***	Support
NE ← PV	−0.322	0.078	−4.108	***	Support
ERB ← PV	0.344	0.074	4.649	***	Support
TR ← PE	0.97	0.077	12.646	***	Support
TR ← NE	−0.093	0.041	−2.3	0.021	Support
ERB ← PE	0.433	0.086	5.031	***	Support
ERB ← NE	0.021	0.039	0.531	0.595	Not support

***p-value < 0.001.

### Mediation effect

SPSS is used in this study to test the mediation effect; if the total effect does not pass through 0 in the 95% confidence interval, the total effect is significant, and vice versa; if the direct effect does not pass through 0 in the 95% confidence interval, the direct effect is significant, and vice versa; and if the indirect effect does not pass through 0 in the 95% confidence interval, the indirect effect is significant. Negative emotions’ independent mediation was not examined since the direct effect of negative emotions on environmentally responsible behavior was not statistically significant (*p* = 0.595 > 0.05), while Hypothesis 11 was rejected.

Results for “perceived value → positive emotion → environmentally responsible behavior” shown in Table 4 demonstrate that the total effect does not pass through 0 at the 95% confidence interval, indicating that the total effect is significant, and the total effect is 0.612; the direct effect does not pass through 0 at the 95% confidence interval, indicating that the direct effect is significant, and the effect is 0.399, accounting for 65% of the total effect. Positive emotions may have partially mediated the effect of perceived value on environmental behavior because the indirect effect did not pass through 0 at the 95% confidence interval, indicating a substantial indirect effect with an effect size of 0.214, accounting for 35% of the total effect.

**Table 4:** Mediation effect.

Path	Effects		SE	LLCI	ULCI		Mediation
PV → PE → ERB	Direct	0.399	0.043	0.315	0.482	65%	Yes
	Indirect	0.214	0.054	0.115	0.328	35%	
	Total	0.612	0.037	0.54	0.685		
PV → PE → TR	Direct	0.39	0.046	0.299	0.48	56%	Yes
	Indirect	0.309	0.036	0.238	0.379	44%	
	Total	0.699	0.043	0.615	0.783		

Results for “perceived value → positive emotion → visitor recollection” shown in Table 4 demonstrate that the total effect did not pass through 0 at the 95% confidence level, indicating that the total effect was significant and having an effect size of 0.699; the direct effect did not pass through 0 at the 95% confidence level, indicating that the direct effect was significant and having an effect size of 0.39, accounting for 56% of the total effect; and the indirect effect did not pass through 0 at the 95% Positive emotions may have partially mediated the effect of perceived value on tourists’ recollection because the indirect effect did not pass through 0 at the 95% confidence interval, indicating that it was significant and accounting for 44% of the total effect. The indirect effect had an effect size of 0.309 and was significant.

## Conclusion and implications

### Conclusion

This study, which was inspired by the S-O-R theory, integrated perceived value, personal positive and negative emotions, and tourist memories to create a research model of visitors’ intention to act sustainably based on their unique experiences at industrial heritage tourism sites. The authentic experience has a significant positive effect on tourists’ perceived value and both positive and negative emotions.

First, The results confirm the proposed effects of authenticity experience on perceived value (H1: *β* = 0.662; CR = 8.176), positive emotions (H2: *β* = 0.413; CR = 6.636) and negative emotions (H3: *β* = 0.336; CR = 3.694), as were the proposed effect of perceived value on positive emotions (H4: *β* = 0.47; CR = 8.365) and negative emotions (H5: *β* = −0.322; CR = −4.108). perceived value of the authentic experience and the site’s embodiment of industrial heritage will result in both positive and negative emotions. It is clear that by stimulating visitors’ authentic experience at industrial heritage tourism sites, different emotions related to personal embodied value will be elicited in them; the more visitors who have strong memories of their travels, the higher the sense of authentic experience at industrial heritage tourism sites.

Second, in line with our stated hypothesis, the results confirm the proposed effects of positive emotions on tourists’ recollection (H6: *β* = 0.97; CR = 12.646), negative emotions has a negative effect on tourists’ recollection (H7: *β* = −0.093; CR = −2.3). The empirical study’s findings indicate that positive emotions have a significant positive effect on tourists’ recollection, whereas negative emotions have a considerable negative effect.

Third, in line with our presented hypothesis, the results confirm the positive emotions has a positive effect on ERB (H8: *β* = 0.433; CR = 5.031), tourists’ environmentally responsible behavior is significantly improved by positive emotion and perceived value. However, contrary to our proposed hypothesis, the negative emotions also has a positive effect on ERB (H9: *β* = 0.021; CR = 0.531), the empirical findings indicate that negative emotions do not significantly affect tourists’ environmentally responsible behavior. A more thorough investigation into the causes of this found that, in some non-hedonic trips, negative emotions not only do not make tourists feel unpositive, but instead are more likely to encourage people to reflect on the meaning of life through such emotions (Martela and Steger, 2016). Visitors to a slum, for instance, change their perspective on material wealth (Zahra and McIntosh, 2007).

They will therefore be more appreciative of their hard-earned life and have a deeper knowledge of what life is all about (Martela and Steger, 2016). According to Martela and Steger’s (2016) study, pilgrimage tourism is a way of life that can enrich one’s quality of life. Similar to that, in this paper’s survey of industrial heritage tourism sites, we discovered a number of negative effects like soil erosion caused by man’s destruction of the once-natural landscape, leading to negative emotions like negativeness and grief in the visitors but also making them more capable of stimulating the development of environmentally responsible behavior.

### Theoretical implications

Few studies have examined the factors that affect the ecologically responsible behavior of authentic experiences in industrial heritage tourism sites, despite the fact that research on authentic experiences in tourism has significantly increased in recent years. The theoretical implications of this study are outlined in the sections below after synthesizing the findings of empirical studies and filling in any research gaps.

First, a thorough framework is constructed based on the S-O-R theory to describe how visitors’ experiences of authenticity of industrial heritage sites lead them to adopt ecologically responsible behaviors. Visitors’ intentions to engage in environmentally responsible behavior are largely influenced by their positive and negative emotions, visitor recollection, and other critical factors.

Second, the findings of the empirical study indicate that the associations between perceived value and visitor recollection as well as perceived value and environmentally responsible behavior are partially mediated by positive emotion. We fill this research gap by further substantiating the mediating function that emotions play in the tourism experience by quantitative methods, even though this is congruent with Tung and Ritchie’s (2011) findings through qualitative research.

Third, this study emphasizes the significance of tourists’ emotions by focusing on the function of both good and negative emotions in the tourism experience. A study of previous research finds that little attention has been paid to tourist emotions, particularly negative emotions, and that earlier studies have concentrated on the effect of visitor satisfaction on behavioral intentions, among other things. This study explores the reasons why negative emotions are more likely to encourage guilt and reflection about the event, and hence push tourists to adopt positive behaviors, in contrast to the premise that negative emotions have a detrimental effect on tourists’ behavior. This study analyzes the significance of emotions (both positive and negative emotions) on tourist behavior and stresses the effect of negative emotion. How to arouse tourists’ emotions has been a hot topic for scholars to explore given that emotions play a significant part in tourism, and this study fills a critical vacuum in the pertinent literature.

Fourth, a thorough examination of the relationship between the authentic experience and visitors’ emotions demonstrates that the majority of earlier studies have concentrated on the effect of variables like perceived services and scenic image on visitors’ emotions, but have not taken into account the fact that the authentic experience of tourism resources also has a significant effect on visitors’ emotions. Few researchers have examined the effect of the authentic experience of tourism resources’ experiences on visitors’ emotions, despite several studies showing that product qualities greatly influence tourists’ emotional responses. This study fills the knowledge gap about the causes of tourism emotions by investigating the effects of the uniqueness of tourism resources on both good and negative emotions experienced by visitors.

### Practical implications

The following management implications for the sustainable development of industrial heritage tourism sites are suggested by this study in terms of specific practices.

First, visitors can generate various perceptual values through the experience of industrial heritage authenticity by fully utilizing the contextual factor of authenticity, and the embodied perceptual values can directly support visitors’ intentions of engaging in environmentally responsible behavior. Because of this, it is advised that industrial heritage tourism scenic spots, on the one hand, should maintain all types of authentic landscapes well on a daily basis; on the other hand, the scenic spots can use cutting-edge technologies like virtual reality to simulate and recreate the ecological landscape of the scenic spots before they were over-exploited, so that visitors can have a sense of contrast and improve their awareness of environmentally responsible behavior. It’s crucial to remember that we should not rely on new technologies excessively to the detriment of tourists’ ability to appreciate nature as it is in its natural state.

Second, by using intra-individual emotions, rational use of positive and negative emotions, as well as memories of tourists, can encourage environmentally responsible behavior. Empirical findings show that memories and emotional experiences, such as good and negative emotion, perceptions of value, have a big effect on environmentally responsible behavior. Destination administrators should therefore develop more memorable tourist experiences for visitors. According to Tung and Ritchie (2011), scenic managers should work to advance the development of the picturesque environment to raise the possibility that visitors will form more lasting memories (p. 1369). On the one hand, by making promoting the beautiful areas more enjoyable, such as by engaging in various nature-related thematic activities, etc., scenic spots can better emphasize the value of harmony between humans and nature. On the other side, by highlighting negative examples, such as by creating some videos of ecological harm caused by human devastation, etc., scenic locations can also assist tourists in developing a better understanding of the ecological effect of human environmental behavior.

Third, tour operators that want visitors to initiate positive behavior should focus on arousing visitors’ emotions in the increasingly cutthroat world of tourism. Positive emotions encourage tourists to engage in positive behaviors, while a small quantity of negative emotions can spur them on. However, it is worthwhile to investigate how to more effectively elicit negative emotions from tourists in order to encourage them to behave positively. Destination operators should concentrate on communicating authenticity and uniqueness of tourism resources to tourists in their advertising efforts since authenticity of industrial heritage experiences can elicit emotional (both positive and negative emotions) responses from tourists. However, managers must take steps to effectively prevent such negative emotions from occurring and lessen the negative emotions that visitors may experience as a result of poor service quality. This is because the homogeneity of tourism resources and the low quality of personnel services can both cause negative emotions.

### Research limitation and future study

Based on the S-O-R theory, this study investigates a new driving mechanism for visitors’ intentions to engage in environmentally responsible behavior in industrial heritage tourism sites. Although the study offers fresh suggestions for encouraging visitors to engage in environmentally responsible behavior in industrial heritage tourism sites, there might be some limitations:

A deeper grasp of the idea of ecologically responsible behavior, for instance, can be categorised in future study. Visitor recollection can be broken down into positive and negative recollection. Positive emotions were significantly and favorably related to visitor recollection, whereas negative emotions were significantly and negatively related to tourists’ recollection, according to the surface level findings. In order to inform related future research, we anticipate that future studies will examine the effect that positive and negative recollection will have on environmentally responsible behavior.

In addition, practically all of the respondents in this study came from cities close to the study’s location because to policies like the COVID-19 epidemic, and the park’s environment changes with the seasons, which has an effect on how valuable visitors are regarded to be as people. According to Gajić et al. (2022b), potential tourists’ perceived fear of COVID-19 infection affects the their visit intention. As a result, it can result in a restriction on how far the study’s findings can be applied. Future surveys could be undertaken across cultures and nations (e.g., domestic visitors vs. overseas tourists) in order to acquire data samples, which would better support the stability of the study’s model. Moreover, according to Gajić et al. (2022a), obtaining COVID-19 certificates of these industrial heritage attractions is likely to reduce the tourists’ fear of pandemic, therefore future studies could explore the effect of COVID-19 certificates on tourists’ authentic experience and Environmentally Responsible Behaviors.

Further studies might additionally reintegrate the model and investigate whether it contains additional components that might affect visitor recollection and environmentally responsible behavior. Changes brought on by visitors can be more clearly seen, and measurements performed using visitor data before and after visiting a location can be compared. Such studies could offer solid confirmation of the causation of variable interactions as well as useful guidance for the environmental sustainability of travel locations.

## Data availability statement

The raw data supporting the conclusions of this article will be made available by the authors, without undue reservation.

## Author contributions

CW developed the methodology, introduction and conclusions, cited examples of industrial heritage tourism and transformation model types, as well prepared the final contents of the article and proofread and revised it. LN reviewed the literature in terms of industrial heritage tourism models. TZ supplemented the literature review of industrial heritage tourism models, proposed extensions to the introduction and conclusions as well as prepared proposed changes to the article in response to the reviews. All authors contributed to the article and approved the submitted version.

## Conflict of interest

The authors declare that the research was conducted in the absence of any commercial or financial relationships that could be construed as a potential conflict of interest.

## Publisher’s note

All claims expressed in this article are solely those of the authors and do not necessarily represent those of their affiliated organizations, or those of the publisher, the editors and the reviewers. Any product that may be evaluated in this article, or claim that may be made by its manufacturer, is not guaranteed or endorsed by the publisher.
